# Wild food plants and wild edible fungi in two valleys of the Qinling Mountains (Shaanxi, central China)

**DOI:** 10.1186/1746-4269-9-26

**Published:** 2013-04-15

**Authors:** Yongxiang Kang, Łukasz Łuczaj, Jin Kang, Shijiao Zhang

**Affiliations:** 1 College of Forestry, Northwest A&F University, 712100, Yangling, People’s Republic of China; 2 Department of Botany and Biotechnology of Economic Plants, Institute of Applied Biotechnology and Basic Sciences, Werynia 502, 36-100 Kolbuszowa, Poland

**Keywords:** Ethnobotany, Ethnomycology, Wild edible plants, Non-timber forest products

## Abstract

**Background:**

The aim of the study was to investigate knowledge and use of wild food plants in two mountain valleys separated by Mount Taibai – the highest peak of northern China and one of its biodiversity hotspots, each adjacent to species-rich temperate forest vegetation.

**Methods:**

Seventy two free lists were collected among the inhabitants of two mountain valleys (36 in each). All the studied households are within walking distance of primary forest vegetation, however the valleys differed in access to urban centers: Houzhenzi is very isolated, and the Dali valley has easier access to the cities of central Shaanxi.

**Results:**

Altogether, 185 wild food plant species and 17 fungi folk taxa were mentioned. The mean number of freelisted wild foods was very high in Houzhenzi (mean 25) and slightly lower in Dali (mean 18). An average respondent listed many species of wild vegetables, a few wild fruits and very few fungi. Age and male gender had a positive but very low effect on the number of taxa listed.

Twelve taxa of wild vegetables (*Allium* spp., *Amaranthus* spp., *Caryopteris divaricata*, *Helwingia japonica*, *Matteucia struthiopteris*, *Pteridium aquilinum*, *Toona sinensis*, *Cardamine macrophylla*, *Celastrus orbiculatus*, *Chenopodium album*, *Pimpinella* sp., *Staphylea bumalda* &*S. holocarpa*), two species of edible fruits (*Akebia trifoliata*, *Schisandra sphenanthera*) and none of the mushrooms were freelisted by at least half of the respondents in one or two of the valleys.

**Conclusion:**

The high number of wild vegetables listed is due to the high cultural position of this type of food in China compared to other parts of the world, as well as the high biodiversity of the village surroundings. A very high proportion of woodland species (42%, double the number of the ruderal species used) among the listed taxa is contrary to the general stereotype that wild vegetables in Asia are mainly ruderal species.

The very low interest in wild mushroom collecting is noteworthy and is difficult to explain. It may arise from the easy access to the cultivated *Auricularia* and *Lentinula* mushrooms and very steep terrain, making foraging for fungi difficult.

## Introduction

Chinese culinary culture is renowned for its use of an extremely large number of ingredients. In many parts of China a large number of wild vegetables is still used, both by peasants in remote rural areas and in restaurants, particularly those located in or near national parks and other high biodiversity areas [1-13], making China one of the best examples of a *herbophilous* country [13,14]. Since antiquity, Chinese scholars have extensively written about the food qualities of wild plants [15]. Research on the potential nutritional qualities of wild food resources and their distribution was carried out in most Chinese agricultural institutions during the 20th century. Although we know much about edible plants, which are used in various parts of China, this knowledge, due to its vast quantity, has still not been properly synthesized [16]. Comparative reviews of the use of wild food resources in different regions of China are also needed. Another interesting issue, little explored, is that of gender and age differences in the use of wild food resources (but see [11,12]).

In the previous paper from this part of the Qinling Mountains the use of wild edible plants and fungi in one relatively isolated mountain valley of the Qinling Mountains was documented [13], giving a detailed list of wild plants used there. As many as 159 species of edible plants and 13 taxa of fungi were recorded. A large proportion of them is still used. The local population has a deep knowledge of these plants and their preparation techniques. Additionally local farmers eat (after special preparation) considerable amounts of *Aconitum carmichaeli* tubers, a plant regarded as one of the most toxic plants on earth [17]. The aim of this study was to compare data from that valley with the use of wild food plants in the neighbouring valley characterized by easy access to the urban centers of the Shaanxi province. We also wanted to look at age and gender differences in the use of wild plants and fungi in these two places.

## Study area

**Figure 1:**
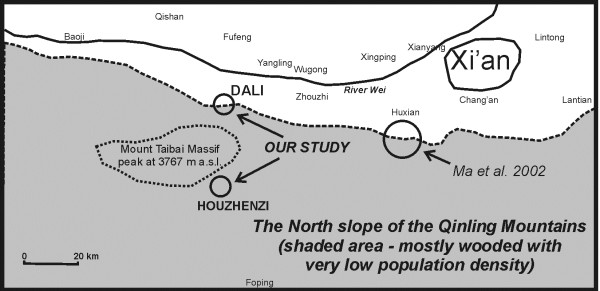
The location of the studied valleys.

The study area was located in the vicinity of the Taibai Nature Reserve, with the highest peak of northern China in the center of the reserve (Mt Taibai 3767 m a.s.l.). The nature reserve protects a highly diverse flora – from warm temperate (with subtropical elements) to alpine at the top – of over 1700 species, which constitutes approximately 60% of the Qinling range flora [18,19] (Figure 1).

Two valleys were chosen for the study. The first valley is located in the Heihe National Forest Park, on the southern edge of the Mount Taibai. The National Forest Park (a less strict protection regime) is the southern extension of the Taibai Nature Reserve, and mainly protects species-rich forests. The area is completely covered by ancient forest vegetation and rocky outcrops. The river Heihe valley belongs to the Houzhenzi administrative unit (town, *zhen*(镇)), with an area of 822 km^2^. It is a very isolated place, which has vehicular access to the county town of Zhouzhi (where the post-office and schools are located) only via a 2.5 h drive through a winding precipitous gorge, often blocked for days by falling rocks. Until 1962 the valley belonged to Foping county. The whole valley is inhabited by 3,500 people – ca. a thousand in the main settlement of Houzhenzi, and the rest in hamlets scattered in the forest. The studied villages lie between 1000 and 1400 a.s.l. At these altitudes the climate is humid temperate, with daily temperatures in summer oscillating around 20-30°C and winter temperatures around 10°C to – 10°C. The mean annual temperature in Houzhenzi is 8.2°C, with a high rainfall of nearly 1000 mm, out of which 44% is concentrated in the summer months [20,21]. The dominant vegetation is the species-rich *Quercus variabilis* and *Q.aliena* var. *acuteserrata* forest, with an admixture of *Pinus tabulaeformis,* and many deciduous tree species (e.g. *Acer* spp., *Tilia* spp.).

The majority of the local population are subsistence farmers who grow maize, potatoes, wheat and beans. The basic staples of the local population are potato, maize and rice. Each farm usually also has chickens and pigs, so eggs, poultry and cured pig meat (*larou*) are frequent components of diet as well. Sources of cash income are the orchards of zaopi (*Cornus officinalis*), walnuts (*Juglans regia*) and northern Sechuan pepper (*Zanthoxylum bungeanum*). Digging out medicinal roots and collecting medicinal herbs for wholesale buyers is also a very popular activity. Many peasant families host tourists (many of them hikers), as part of the agritourist farm system called *nongjiale* (农家乐)*.* A certain influx of tourists in the valley is caused by the fact that it lies on a picturesque and wild foot trail to Mount Taibai.

The second valley, later called Dali valley (after the largest village in it) is located on the northern edge of the Taibai Mt. It is less isolated than the former valley, being easily accessible by car from the county town Meixian, Xi’an and other cities in central Shaanxi. It belongs to the Meixian county, Yingtou administrative unit (town, *zhen* (镇)), with 11 villages, ca. 20 thousand inhabitants and 202 km^2^). The actual valley we studied has about 1500 inhabitants and an area of 21 km^2^. The studied villages lie between 700 and 1200 a.s.l. There is no meteorological data on the climate of the area. The mean temperature from Meixian County weather station (alt. ca. 550 m), 15 km from Dali, is about 12.9°C, so we estimate the mean temperature in Dali valley as 8 – 11°C, depending on elevation and location [22,23]. The dominant vegetation is the species-rich *Quercus variabilis* and *Q.aliena* var. *acuteserrata* forest, with an admixture of *Pinus tabulaeformis,* and many deciduous tree species (e.g. *Acer* spp., *Tilia* spp., *Platycladus orentalis*, *Sorbus* spp., *Litsea* spp.etc.).

The majority of the local population are subsistence farmers who grow maize, potatoes, wheat, and beans. Sources of cash income are walnut orchards (*Juglans regia*), kiwi fruits (*Actinidia chinensis*) and northern Sechuan pepper (*Zanthoxylum bungeanum*). Digging out medicinal roots and collecting medicinal herbs for wholesale buyers is much less important than in the Heihe valley, although a large company buying herbs from all over the central Qinling is located there. A number of stone processing businesses operate in the largest villages. Tourism is little developed and, in contrast to the Houzhenzi valley, there are very few *nongjiale* in the valley (in contrast to it a neighbouring valley has one of the main entrances to the Taibai Reserve (Red Valley Entrance) and experiences much tourism, but we did not study it due to the large proportion of outsiders who settled there).

Both valleys are inhabited by people of Chinese Han nationality. Most inhabitants are local, although some individuals are outsiders who (or whose families) settled, escaping mid-20th century famines from densely populated parts of Shaanxi and Sichuan, or migrated later due to the socio-cultural situation in China. They speak the Shaanxi dialect of Mandarin (Guanzhong dialect, a form of Zhongyuan dialect). The inhabitants of the Dali valley speak a standard form of the Guanzhong dialect, whereas in the Houzhenzi valley, which is more southern, the influence of the Sichuan dialect is visible [24-26]. A detailed description of the economic status of villages in a neighbouring valley of Qinling Mountains, also applicable to the study area, was given by Neurauter et al. [27].

## Methods

The field research was conducted in June and July 2011, as well as in August 2012, using structured freelisting interviews (36 freelists were created in each valley). The listed taxa were identified using transect walks and cross-checking of the gathered herbarium specimens. Participant observation and long semi-structured interviews with key informants were also used to establish the role of wild food in the local communities.

The research was carried out following the code of ethics of the American Anthropological Association [28] and the International Society of Ethnobiology Code of Ethics [29] and general standards of collecting ethnobiological data presented by major ethnobotany textbooks [30-33]. Oral prior informed consent was acquired.

In the Houzhenzi valley the interviewees came from the following villages: Houzhenzi, Diaoyutai, Huaerping, Jiangjiaping and Sanhe. The mean age of participants was 50 (median 49.5, aged from 16 to 83; 20 women and 16 men). In the Dali valley we interviewed people living in: Dalicun, Dawancun, Shapocun, Fufeng, Honghecun, Lijiahecun, Liguancun and Tangyu (21 women and 15 men). The mean age of participants was 58 (median 59, age from 27 to 84). During freelisting we separately asked, which species of wild vegetables (including underground organs), wild fruits and wild mushrooms were used. Making three separate freelists enabled the comparison of the use of these categories and helped elicit answers from the respondents [34,35]. Freelists were made orally and written down on the spot by our team, including the Chinese-script version of the plant/fungi names, which was available to the interviewees.

The study started from a few informants found using the snowball technique, but most interviewees were found by systematic walks through the village, visiting houses and asking the inhabitants if they wanted to take part in the study. We usually interviewed only one person from each household, only occasionally were two people from the same house interviewed, if there were signs that their knowledge differed (e.g. one of the spouses comes from another village, etc.). In a few cases free listing was done in the presence of other family members or neighbours, but one person, delegated as the most knowledgeable, was the main interviewee. Voucher specimens are stored in the Department of Forestry, Northwest A&F University in Yangling.

A Spearman rank correlation matrix was calculated for all the variables studied. Additionally the Mann-Whitney U test was used to test differences between groups (male versus female population, Houzhenzi valley versus Dali valley). Unfortunately the distribution of variables was not normal, even after log-transformation, so we could not perform a multi-factor ANOVA analysis. An open access statistical program, PAST [36,37], was used for statistical analyses.

## Results

### General figures

**Figure 2:**
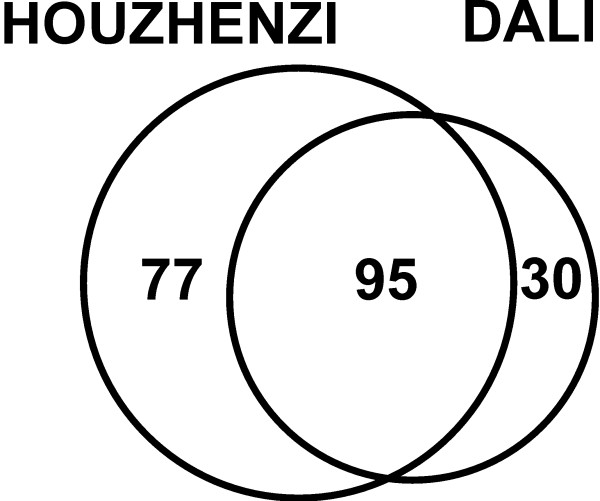
The overlap between the number of species of plants and mushrooms used in both valleys.

Altogether 167 folk plant taxa with 185 species from 72 families and 17 fungi folk taxa (out of which we identified 12 taxa to genus or species level) were listed by the informants. This includes 126 species of green vegetables, 25 species with edible roots/rhizomes/tubers/bulbs, five species of flowers, 42 with edible fleshy fruits and four of dry fruits/seeds (Figure 2). In Houzhenzi 158 plant species and 14 fungi taxa were mentioned by the informants as eaten at least once in their lifetime, but only 130 plant species and 13 fungi species were confirmed as eaten by more than one person (Tables 1,2 and 3). In Dali 113 plant species and 12 fungi taxa were mentioned by the informants as eaten at least once in their lifetime, but only 77 plant species and 11 fungi taxa were confirmed as eaten by more than one person. There was a considerable overlap in the species listed in both valleys (Figure 2).

**Table 1:** Rank correlation matrix (Spearman rho coefficient)

	**Age**	**Male gender?=?1**	**Houzhenzi valley?=?1**	**No of all species**	**No of vegetable species**	**No of fruit species**
Male gender?=?1	-0.34**					
Houzhenzi valley?=?1	-0.25*	0.028				
No of all species	0.16	0.23	0.31**			
No of vegetable species	0.26*	0.27*	0.35**	0.89***		
No of fruit species	-0.025	0.029	0.089	0.68***	0.35**	
No of fungi	-0.0009	0.20	0.14	0.46***	0.28*	0.34**

*** p?<?0.001; ** p?<?0.01; * p <0.05; values without asterisks are not statistically significant.

**Table 2:** Basic features of plant and fungi use in the Qinling mountains

	**No. of species used**	**Frequency of use**	**Consumption after boiling or stir-frying or in soup**	**Raw consumption**	**Drying for further use**	**Lacto-fermenting**	**Gender differentiation**	**Age differentiation**
Wild vegetables	high	high	yes	no	frequently	rarely, more often in the past	men know slightly more	low
Wild fruits	intermediate	low	no	yes	never	no	no	insignificant
Wild fungi	low	low	yes	no	very rarely	no	men know slightly more (?)	insignificant

**Table 3:** **Wild food species used in the northern slope of the Qinling mountains (plant families given according to APGIII **[69]**)**

**Family**	**Name**	**Part used for food**	**Habitat**	**Frequency in Houzhenzi**	**Frequency in Dali**	**Name in Houzhenzi**		**Name in Dali**		**Ma**	**Voucher specimen no.**
	VASCULAR PLANTS										
Actinidiaceae	Actinidia chinensis Planch.	fr	f	***	***	yemihoutao	野猕猴桃	yemihoutao	野猕猴桃		193
	Actinidia polygama Franch. & Sav.	fr	f		1			gezaomihoutao	葛枣猕猴桃		
Amaranthaceae	Achyranthes bidentata Blume	ap	e	*		niuxi	牛膝				185
	Amaranthus caudatus L.	ap	r	**	1	tianximi	天西米	ximi	菥蓂		
	Amaranthus retroflexus L., A. paniculatus L., A. viridis L. etc.	ap	r	****	****	hancai, renhancai	汉菜, 人汉菜,	hancai, renhancai	汉菜, 人汉菜,		56, 150
	Chenopodium album L., Chenopodium giganteum D. Don	ap	r	****	***	huihuicai	灰灰菜	huihuicai	灰灰菜	x	49, 160, 216
	Chenopodium glaucum L.	ap								x	
	Kochia scoparia (L.) Schrad.	ap	e	*	yes	tiesaoba	铁扫把	dudusaozhou	独独扫帚		118, 218
Amaryllidaceae	Allium funckiacfolium Hand.-Mazz.	ap								x	
	Allium senescens L.	ap	e							x	
	Allium ovalifolium Hand.-Mazz., Allium cf victorialis L.	ap, u	e	*	*	gejiu, yejiu	茖韭, 野韭	gejiu	茖韭		200
	Allium paepalanthoides Airy Shaw	ap, u	e	***	1	tiansuan	天蒜	tiansuan	天蒜		27
	Allium spp. (Allium cf. senescens L.; Allium macrostemon Bunge)	ap	e	****	****	aijiucai, aisuan, yesuan, yongbaotou, luoerjiu, zongbaotou, yejiucai	崖韭菜, 崖蒜, 野蒜, 罗儿韭,棕包头, 野韭菜	xiaosuan, luoerjiu, yancong, aisuan	小蒜, 罗儿韭, 岩葱, 崖蒜		201, 202, 229
	Allium tuberosum Rottler ex Spreng.	ap	e		1			yejiucai	野韭菜		227
Anacardiaceae	Rhus potatninii Maxim.	ap	f	1	*	wubeizishu	五倍子树	wubeizi	五倍子		
	Rhus verniciflua Stokes	ap	f	*	1	qishu	漆树	qishuya	漆树芽		18
Apiaceae	Cryptotaenia japonica Hassk.	ap	f	*		yajiuban	鸭脚板				103, 171
	Daucus carota L.	ap	r							x	
	Ligusticum sinense Oliv. 'Chuanhsiung '	ap	r	*	1	chuanxiong	川芎	chuanxiong	川芎		51
	Ligusticum levisticum L.	ap	r		1			gaoben	藁本		
	Oenanthe javanica DC.	ap	x	1	**	beizhe	背折	shuiqincai	水芹菜	x	65, 213
	Pimpinella sp.	ap	f w	****		shuiqincai, shaqincai	水芹菜, 沙芹菜				105, 159
	Tongoloa silaifolia (de Bois.) Wolff	ap	e		1			taibaisanqi	太白三七		
Araliaceae	Acanthopanax gracilistylus W.W.Sm.	ap	f							x	
	Aralia chinensis L.	ap	f	**	**	cilongpao	刺龙袍	cilongpao, cichuntou, laohanchuizi	刺龙袍, 刺椿头, 老汉锤子	x	2
Aristolochiaceae	Asarum himalaicum Hook.f. & Thomson ex Klotzsch	ap, u	f	*		maoxixin	毛细辛				7
	Asarum sieboldii Miq.	ap, u	f	*		xixin	细辛				24, 163
Asclepiadiaceae	Cynanchum giraldii Schltr.	u	f	*		geshanxiao	隔山消				133
Asparagaceae	Polygonatum cyrtonema Hua	u	f	1		huabeimaoqi	华北毛七				74
	Polygonatum megaphyllum P.Y.Li and Polygonatum odoratum L.	u	f	*		yuzhu, yuzhushen	玉竹, 玉竹参				31, 34
	Smilacina japonica A.Gray , Smilacina henryi (Baker) Hara	ap	f	*	*	piantoucai	偏头菜	piantoucai	偏头菜		6, 129
Asteraceae	Carduus crispus L.	ap								x	
	Anaphalis aureopunctata f.flavescens Lingelsh et Borza	ap	e	*		shiqucao	鼠曲草				137
	Anaphalis margaritacea Benth. & Hook.f.	ap	e	*		qingmingcai	清明菜				116, 161
	Arctium lappa L.	ls, u	x	*		niubangzi	牛蒡子			x	23, 156
	Artemisia sacrorum Ledeb.	ap								x	
	Artemisia argyi H.Lév. & Vaniot	ap								x	
	Artemisia capillaris Thunb.	ap								x	
	Artemisia subdigitata Mattf.	ap	r	*	*	ai	艾	shuihao	水蒿		21
	Cacalia roborowskii (Maxim.) Y.Ling	ap	e	*		xiongerduo	熊耳朵				44
	Cirsium arvense var. setosum (Willd.) C.A.Mey	ap	r	**	**	honghuamiao, ciji	红花苗, 刺蓟	ciji	刺蓟		178, 210
	Cirsium spp. eg Cirsium botryoides Petrak ex Hand.-Mzt.	ap	r	*		xiaoji	小蓟			x	62, 101, 117
	Cirsium segetum Bunge	ap	r								
	Conyza canadensis (L.) Cronquist	ap	r	1		guangguangcao	冠罐草				141
	Erigeron acer L.	ap	r	1		guangguangcao	冠罐草				59
	Hieracium sp.	ap	r	**		kuma(i)cai	苦荬菜				197
	Ixeris denticulata (Houtt.) Stebbins	ap	r							x	
	Ixeris chinensis Nakai.	ap	f							x	
	Ixeris sonchifolia Hance	ap	r	***	***	kumaicai	苦荬菜	kuqu		x	122
	Kalimeris pinnatifida (Maxim.) Kitam.	ap	e	*		malantou	马兰头				52, 135, 180
	Lactuca serriola L.	ap	r	**		xiaobaijiang, xiaokumacai, kumacai	小苦荬菜, 苦荬菜				107
	Leontopodium japonicum Miq.	ap	e	*		shuqucao	鼠曲草				136
	Picris hieracioides L.	ap	r	*		kumaicai	苦荬菜				123, 181
	Saussurea dolichopoda Diels	ap	f	***	***	kongtongcai, kongxincai	空筒菜, 空心菜	xiangtongcai, kongxincai	响筒菜, 空心菜		177, 235
	Senecio scandens Ham.	ap				jiuliming	九里明				
	Sinacalia tangutica (Maxim.) B.Nord.	u	e f	*	*	shuiluobo	水萝卜	shuiluobo	水萝卜		43, 151
	Sonchus asper L	ap	r							x	
	Sonchus oleraceus L.	ap	r							x	
	Sylibum marianum L.	ap								x	
	Taraxacum mongolicum Han.-Mzt	ap	r	**	**	pugongying, kumaicai, dakucai	蒲公英,苦荬菜,大苦菜	pugongying	蒲公英	x	8
Balsaminaceae	Impatiens notolopha Maxim.	ap	f	*		daolaonnen	到老嫩				179
Begoniaceae	Begonia sinensis A.DC.	ap	f	*		yikouxie	一口血				138
Brassicaceae	Capsella bursa-pastoris Medik.	ap	r	***	*	didicai	地地菜	diercai, didicai	地儿菜, 地地菜	x	25
	Cardamine engleriana O.E.Schultz.	ap	w		1			guangtoushansuimiji	光头山碎米荠		
	Cardamine macrophylla Willd.	ap	f w	***	****	shijiacai	石夹菜	shijiacai	石夹菜		20
	Cardamine spp. (other smaller species e.g. Cardamine flexuosa With., C. hirsuta L.)	ap	f	*	*	xiaoshijiacai	小石夹菜	huadidi, huadier	花地地(small caidamine)		228
	Descurainia sophia (L.) Webb ex Prantl	ap	e	**		yinchen, mihao	因陈 , 米蒿				142
	Rorippa montana Small	ap	r	*	**	manjingcai, lalacai	蔓茎菜, 辣辣菜	lalacai, lazicai	辣辣, 辣子菜	x	234
	Rorippa indica L.	ap	r							x	
	Thlaspi arvense L.	ap	r	***	*	jidanhuang	鸡蛋黄	kugen	苦根		10, 228
Campanulaceae	Adenophora spp. (Adenophora capillaris Hemsl., Adenophora polyantha Nakai)	ap, u	e	***	***	naijiangcai	奶浆菜	naiercai, nainaicai	奶儿菜, 奶奶菜		134, 198, 199, 226
Caprifoliaceae	Lonicera standishii Carr.	fr	f	*	**	kutangpao	苦糖泡	yangnaizi, kutangpao	羊奶, 苦糖泡		26
	Sambucus williamsii Hance	ap	f	1	1	jiegumu	接骨木	shuhuacai	树花菜		47
	Viburnum sargentii Koehne	fr	f	1		[no name]					99
Caryophyllaceae	Arenaria serpyllifolia L.	ap								x	
	Lychnis senno Siebold & Zucc.	ap	e	*		honghuacai	黄花菜				57, 186
	Silene conoidea L.	ap	r	*	**	maipiancai	麦片菜	maihuaping	麦花瓶	x	140
	Stellaria media (L.) Vill.	ap	r	*		eerchang	鹅儿肠				33, 152
	Vaccaria segetalis (Neck.) Garcke	ap	r		1			pangwawa	胖娃娃		
Celastraceae	Celastrus orbiculatus Thunb.	ap	f	****	***	baiwanye	白蔓叶	baiwanye	白蔓叶		17
	Euonymus alatus (Thunb.) Siebold	fr	f	1	1	bashu, bamu	巴树, 巴木	bamu	巴木		
	Parnassia wightiana Wall.	ap	f	1		xinyecao	心叶草				147
Cephalotaxaceae	Cephalotaxus sinensis (Rehder & E.H.Wilson) H.L.Li	fr	f	*	1	baigeiguo, bizishu, shuibai, sunguo	白盖果,篦子树,水柏,松果	sanjianshanguo	三尖杉果		14, 164
Commelinaceae	Commelina communis L.	ap	r	*		danzhuye, zhuyecao, miandazi	淡竹叶,竹叶草,面鞑子				106, 158
Convallariaceae	Tricyrtis macropoda Miq.	ap	f	***	***	huangguacai	黄瓜菜	huangguacai	黄瓜菜		4, 176
Convolvulaceae	Calystegia hederacea Wall.	ap	r		*			dawanhua, labahua	打碗花, 喇叭花	x	
	Cuscuta cf chinensis L.	ap	e		1			wugencao	无根草		
Cornaceae	Cornus kousa Bürger ex Miq.	fr	f	***	*	shizao	石枣	shizaozi, yelizhi	石枣子, 野荔枝		16, 165
Corylaceae	Corylus heterophylla Fisch. ex Besser	fr	f	*	1	zhenzi, maoli, maolizishu,	榛子, 毛栗子树	zhenzi	榛子		15, 167
Crassulaceae	Sedum aizoon L., S. sarmentosum Bunge, pampaninii Raym.-Hamet, S. lineare Thunb.	ap	e	**	**	gouyaban, gouzacai, machijie, dabusi, chuipencao	狗牙瓣, 打不死	shitouya, gouyacai, manaocai	石头芽, 狗牙菜, 玛瑙菜		75, 131, 120, 127, 153, 192, 222
	Sedum amplibracteatum K.T.Fu	ap	f	***	***	huaqiaoman, lazimiao, lajiaomiao, yelacai	花荞蔓, 野辣子苗苗, 辣椒苗, 叶辣菜	lajiaomiao, lalacai, lazicai, yelazi	辣椒苗, 辣子菜, 野辣子		168, 182
	Sedum verticillatum L.	ap	e		1			jingtiansanqi	景天三七		
Cucurbitaceae	Gynostemma pentaphyllum (Thunb.) Makino	ap	e	1		jiaogulan	绞股蓝				no
Dennstaedtiaceae	Pteridium aquilinum (L.) Kuhn	ap, u	x	****	****	juecai, juegen, longzhua	蕨菜, 蕨根, 龙爪菜	yangjuecai, yangjuegen	羊蕨菜, 羊蕨根	x	9, 214
Dioscoreaceae	Dioscorea batatas Decne.	u	f	*	*	shanyao	山药	yeshanyao	野山药		53, 191, 209
Dryopteridaceae	Cyrtomium fortunei J.Sm.	ap								x	
	unidentified fern cf. Dryopteridaceae	ap	f	1		xiaojitoucai	小鸡头菜				111
Ebenaceae	Diospyros lotus L.	fr	x		*			shishu, junqianzi	柿树, 君迁子		
Eleagnaceae	Elaeagnus umbellata Thunb.	fr	e	***	1	yangnaizi, niunaizi	羊奶子, 牛奶子	jianzi	剪子		29, 232
	Hippophae rhamnoides L.	fr	x		1			xiaoguoshaji	小果沙棘		
Ericaceae	Pyrola decorata Andres	ap	f	*		hongru, shoucha	红茹,寿茶				68
	Pyrola rotundifolia L.	ap	f	*		bairu, shoucha	白茹,寿茶				69
Fabaceae	Cercis chinensis Bunge	ap	f		1			momoye	馍馍叶		208
	Kummerovia stipueacea (Makim.) Makino	ap	e					qiabuqi	掐不齐		
	Medicago sativa L.	ap	r	*		muxicai	苜蓿菜				54
	Pueraria lobata (Willd.) Ohwi	u	e	**	*	gegen	葛根	gegen	葛根		
	Robinia pseudoacacia L.	ap	f	*	1	huaihua	槐花	huaihua	槐花	x	40
	Vicia cracca L.	ap	r	*		yewandoujian	野豌豆尖				3, 71
	Vicia sp.	ap	r		1			maoshaozi	毛苕子		
Fagaceae	Castanea mollissima Blume	fr	f	**	**	yemaoli, yebanli	野毛栗, 野板栗	yemaoli, yebanli	野毛栗, 野板栗		130
	Quercus variabilis Blume	fr	f	1		xiangzishu	橡子树				
Grossulariaceae	Ribes glaciale Wall.	fr	f	1		[no name]					37
Helwingiaceae	Helwingia japonica (Thunb.) F.Dietr.	ap	f	****	****	yeshanghua	叶上花	yeshanghua, yeshanhua	叶上花, 叶扇花	x	22, 175
Juglandaceae	Juglans cathayensis Dode	fr	f	**	**	yehetao	野核桃	yehetao	野核桃		
Lamiaceae	Caryopteris divaricata Maxim.	ap	f	****	1	choulaohan, laohanxiang	臭老汉/老汉香	choulaohan	臭老汉		109
	Clerodendrum trichotomum Thunb.	ap	f	*		choumudan, choulaohan	臭牡丹,臭老汉				11
	Lycopus lucidus Turcz. ex Benth.	ap	e	*		yebaicai, zelan	野白菜,泽兰				98
	Mentha haplocalyx Briq.	ap	e	1		bohe, yuxiangcao	薄荷,鱼香草				114
	Stachys affinis Bunge	u	r	*		diguniu	地牯牛				67, 157
Lardizabalaceae	Akebia trifoliata (Thunb.) Koidz.	fr	f	***	****	bayuegua, bayuezha	奶浆菜, 八月炸	bayuegua, bayuezha	奶浆菜, 八月炸		119
	Decaisnea fargesii Franch.	fr	f	***	***	maoshigua, yexiangjiao	猫屎瓜, 野香蕉	yexiangjiao, maoshigua, maoershi	野香蕉, 猫屎瓜, 猫儿屎		173
Liliaceae	Hemerocallis spp. (Hemerocallis dumortierii C.Morren, Hemerocallis fulva L.)	fl	e	**	*	yehuanghua	野黄花	yehuanghua	野黄花		42, 139
	Lilium brownii F.E.Brown ex Spae	u	f							x	
	Lilium giganteum Wall.	u	w,f	**		shuibaihe	水百合				35
	Lilium longiflorum Thunb.	u								x	
	Lilium lancifolium Thunb. (as L. tigrinum)	u	x,w	**		yebaihe	野百合				64, 124, 184
Linnaeaceae	Abelia engleriana Rehder	ap	f	***	*	shenxiandoufu	神仙豆腐	shenxiandoufu	神仙豆腐		36
Malvaceae	Grewia biloba G.Don. Var. Parviflora (Bge) Hand.-Mzt.	fr	e		1			gebengbeng	咯嘣蹦		
	Malva sinensis Cav.	if	r	1	1	dawanhua	打碗花	yejinkui	野锦葵		162, 221
Meliaceae	Toona sinensis (Juss.) M.Roem.	ap	e	****	****	xiangchun	香椿	xiangchun	香椿	x	5
Menispermaceae	Cocculus trilobus (Thunb.) DC.	ap	x		1			heimanye	黑蔓叶		233
Moraceae	Broussonetia papyrifera (L.) Vent.	ap, fl	r	*	*	goushuguo, gouye	构树果,构 叶	goutao	构桃	x	132
	Morus australis Poir.	fr	f	*		sangpao, sangshu	桑泡,桑树				45
Onocleaceae	Matteucia struthiopteris (L.) Tod.	ap	x	****	****	jitoucai	鸡头菜	jiwacai, jiercai	鸡娃菜, 鸡儿菜		46, 174, 220
Orchidaceae	Bletilla striata Rchb.f.	u	f	1		baiji	白芨				206
	Gastrodia elata Blume	u	f		1			tianma	天麻		
Oxalidaceae	Oxalis spp. (O. griffithii Edgew. & Hook.f., O. corniculata L.)	ap	f r	*	1	suancao, suancai, suansuancao	酸草,酸菜,酸酸草	suanjiji	酸唧唧		13, 55, 183
Penthoraceae	Penthorum chinense Pursh.	ap	f							x	
Phytolaccaceae	Phytolacca esculenta Van Houtte	ap, u, fr	r		*			jiangliusheng	江柳绳	x	212
Plantaginaceae	Plantago asiatica L.	ap	r e	*		kaimenye, cheqiancao, cheqianzi	开门叶,车前草,车前子				1, 149
	Veronica didyma Tenore	ap	r							x	
Poaceae	unidentified Bambusae	ap	f	1		zhusun	竹笋				
Polygonaceae	Fagopyrum gracilipes (Hemsl.) Dammer	ap	r	1		yeqiaomai, qiaomaimiao	乔麦苗				143
	Polygonum aviculare L.	ap	r e	1	1	bianxu	萹蓄	bianxucai	萹蓄草		63, 219
	Polygonum ciliinerve (Nakai) Ohwi	u	e	*		qiaomaitou	荞麦头				
	Rumex crispus L.	ap	e	**	1	niushetou, yedahuang	牛舌头,野大黄	luerduo	驴耳朵		66
Portulaccaceae	Portulacca oleracea L.	ap	r e		1			machixian	马齿苋	x	
Primulaceae	Lysimachia hemsleyana Maxim. ex Oliv.	ap	e	1		guoluhuang	过路黄				144
Ranunculaceae	Aconitum carmichaelii Debeaux (more often cultivated)	u	x	1	*	wuyao	乌药	wuyao	乌药		50, 155
Rhamnaceae	Berchemia sinica Schneid.	fr	f	*		yaguteng	亚古藤,勾儿茶				121
	Hovenia dulcis Thunb.	fr	f		1			guaizao	拐枣		
	Zizyphus jujuba var. spinosa (Bunge) Hu	fr	e	1				shanzao	山栆		
Rosaceae	Crataegus hupehensis Sarg.	fr	f	*	**	yeshanzha	野山楂	yeshanzha, mianli, mianlizi	野山楂, 面梨, 面梨子		
	Fragaria spp. (Fragaria corymbosa Losinsk., Fragaria pentaphylla Losinsk.)	fr	e	***	*	caomei, dipao, didipaoxiangpao	草莓, 地泡, 地地泡, 香泡	caomei, yecaomei	草莓, 野草莓		58, 169, 170
	Malus prunifolia (Willd.) Borkh.	fr	f		1			qiuzi	秋子		225
	Potentilla indica (Andrews) Th.Wolf	u	r e		*			shemei	蛇莓		217
	Potentilla sp.	ap	r	*		guanyincha	观音茶				115
	Prunus armeniaca L.	fr	x	**	**	yexing	野杏	yexing	野杏		
	Prunus canescens Bois, P. pilosiuscula Koehne	fr	f	**		yeyingtao	野樱桃				39
	Prunus davidiana Franch.	fr	x	1		shantao	山桃				
	Prunus cfr polytricha Koehne	fr	f	*		chuantao	川桃				28
	Prunus persica (L.) Batsch	fr	f	**	***	yetaozi	野桃子	yetaozi	野桃子		38
	Prunus salicina Lindl.	fr	x	***	***	yelizi, zemaili, huolizi, huoli, yemaili	野李子, 火李子, 火李, 野麦李	yelizi, kuli, huoli	野李子, 苦李, 火李		187
	Prunus tomentosa Thunb.	fr	f		***			yeyingtao, maoyingtao, maotao	野樱桃, 毛樱桃, 毛桃		224
	Pyrus xerophila T.T.Yu	fr	e x	***	**	yeli, mali, shanli	野梨, 麻梨, 山梨	yeli, mali	野梨, 麻梨		
	Pyracantha fortuneana (Maxim.) H.L.Li	fr	e	1		jiubingliang	救兵粮				
	Rosa omeiensis Rolfe	fr	e	1		cishiliu	刺石榴				
	Rosa sp.	ap	f	*		cimeihua	刺玫花				110
	Rubus spp.	fr	e x	***	*	duanyangpao, xuangouzi	端阳泡, 悬钩子	meizi	莓子		
	Rubus coreanus Miq.	fr	e	**		cipao, dipao, fupenzi	刺泡, 地泡,覆盆子				30
	Rubus flosculosus Focke	fr	e	**		caizipao	菜子泡				195
	Rubus pungens Cambess.	fr	e x	**		huangcipao	黄刺泡				61
Rubiaceae	Galium aparine L.	ap	r		1			ranwawa	然娃娃		
Rutaceae	Zanthoxylum bungeanum Maxim.	ap, fr	f	*	1	yehuajiao	野花椒	yehuajiao	野花椒		no
Sabiaceae	Sabia shensiensis H.Y.Chen	ap	f	*		qingtengcai, tengercai	青藤菜, 藤儿菜	qingtengwan	青藤蔓		166
Salicaceae	Salix cf babylonica L.	ap	e		1			liushu	柳树		
Santalaceae	Buckleya henryi Diels	fr	f		*			mainhuli, mimianwong	面核梨, 米面翁		231
Saururaceae	Houttuynia cordata Thunb.	ap	r		1			yuxingcao	鱼腥草	x	
Saxifragaceae	Bergenia scopulosa T.P.Wang	ap	w	1		yebaicai	野白菜				
	Chrysosplenium biondianum Engl.	ap	f	***	***	hongjincai	红筋菜	hongjincai	红筋菜		188
	Chrysosplenium sinicum Maxim.	ap								x	
Schisandraceae	Schisandra sphenanthera Rehder & E.H.Wilson	fr	f	****	****	wuweizi	五味子	wuweizi	五味子		48, 196
Solanaceae	Physalis alkekengi L.	ap	r		*			guajindeng, denglonghuacai	挂金, 灯笼花菜		215
	Solanum nigrum L.	ap	r	1	*	suanjiang	酸浆	heilaopo, longkai	黑老婆	x	70, 211
Staphyleaceae	Staphylea bumalda DC., S. holocarpa Hemsl.	ap, fl	f	****		shuhuacai	树花菜				12, 189, 190
Ulmaceae	Ulmus bergmanniana C.K.Schneid., Ulmus propinqua Koidz.	ap, b, if	f e	**	*	yushu	榆树	yushu	榆树	x	32, 60
Urticaceae	Boehmeria gracilis C.H.Wright	ap	x	*		honghema	红河麻				128, 194
	Boehmeria tricuspis Makino	ap	x	*		hema	河麻				76
	Pilea mongolica Wedd.	ap	f	*		daolaonen	到老嫩				207
	Urtica fissa E.Pritz. ex Diels	ap	x	*		baihema	白河麻				41
Violaceae	Viola cf. grypoceras A.Gray	ap	r	1		didingcao	地丁草				145
Vitaceae	Vitis ficifolia Bunge	fr	f	***	***	yeputao	野葡萄	yeputao	野葡萄		19
	FUNGI										
Auriculariaceae	Auricularia sp. (more often cultivated)	fb	f	*	*	muer	木耳	muer	木耳		
Boletaceae	Boletus spp.	fb	f	**	*	niuganjun, dajiaogu	牛肝菌, 大脚菇	niuganjun	牛肝菌		204, 236
Cantharellaceae	Cantharellus cibarius Fr.	fb	f	**	**	huangsijun	牛肝菌	huangsijun, jiyoujun	牛肝菌, 鸡油菌		203
Gomphaceae	Ramaria spp.	fb	f	***	*	shuabajun	刷把菌	guoshuajun	锅刷菌		205
Hericiaceae	Hericium sp.	fb	f	*	*	houtoujun	猴头菌	houtoujun	猴头菌		
Marasmiaceae	Lentinula edodes (Berk.) Pegler (more often cultivated)	fb	f	**	1	yexianggu	野香菇	yexianggu	野香菇		
Meripilaceae	Grifola umbellata (Pers.) Pilát	fb	f	**	**	zhulingjun	猪苓菌	zhulingjun, zhulinghua	猪苓菌, 猪苓花		223
Morchellaceae	Morchella sp.	fb	f	*		yangquejun	羊雀菌				
Pleurotaceae	Pleurotus sp.	fb	f	***	*	dongjun	冻菌	dongjun	冻菌		
Polyporaceae	Laetiporus sulphureus (??)	fb	f	*		jiguanjun	鸡冠菌				
Tricholomataceae	Tricholoma matsutake (S. Ito & S. Imai) Singer (?)	fb	f		*			songrongjun	松茸菌		
	UNIDENTIFIED MUSHROOM	fb	f	*		qiaomianjun	荞面菌				
	UNIDENIFIED TERRESTRIAL GILLED MUSHROOM	fb	f	**	**	banlijun	板栗菌	banlijun, maolijun	板栗菌, 毛栗菌		
	UNIDENTIFIED MUSHROOM	fb	f	**		qiaomaijun	荞麦菌				
	UNIDENTIFIED MUSHROOM	fb	f	1		baogujun	包谷菌				
	UNIDENTIFIED MUSHROOM	fb	f		*			yangdujun	羊肚菌		
	UNIDENTIFIED MUSHROOM	fb	f		*			mabojun	马脖菌		

Ma – Recorded by Ma et al. 2002 [38,39].

Habitat types: e – forest edges, shrubland, forest clearings, grassland; f – forest; r – ruderal; w – water edges; x – ubiquitous.

Parts consumed: fr – fruit, ap –aerial parts, u – underground parts, fl – flowers, b – bark, fb – fruiting body, ls – leaf stalks, if – immature fruits.

Frequency: **** > 50% of respondents; *** > 1/4 of respondents; ** > 1/8 of respondents; * from 2 to 8 respondents; 1 – one respondent.

**Figure 3:**
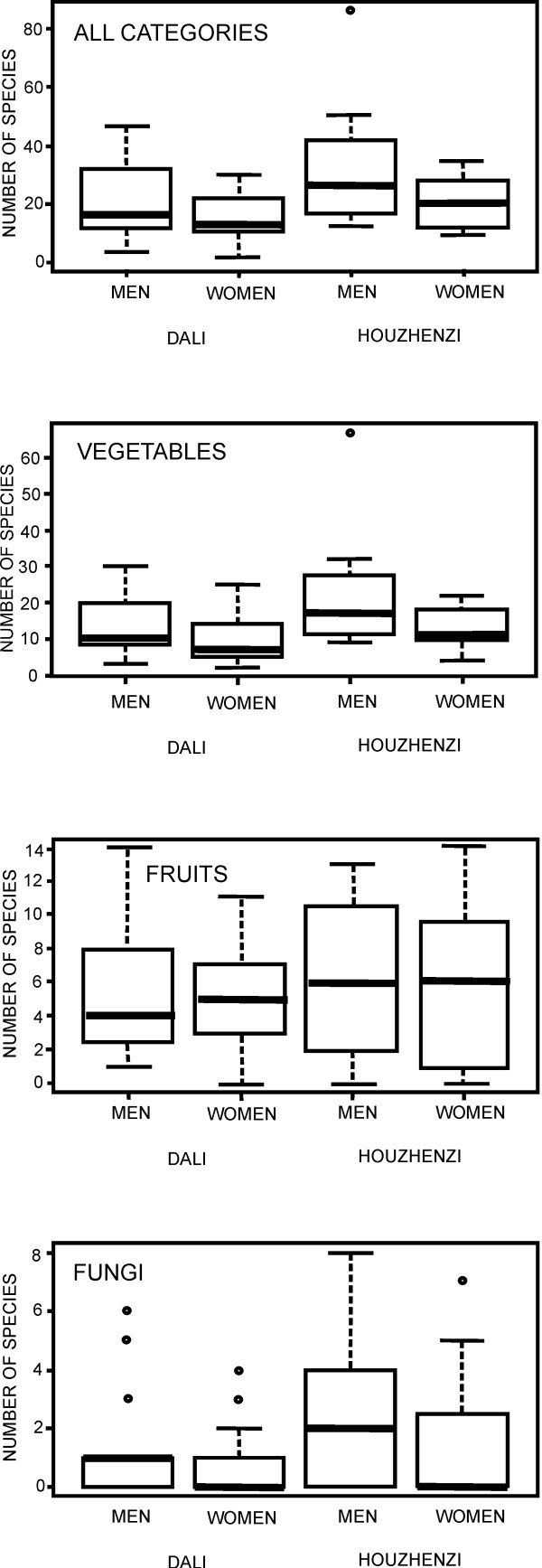
**Species numbers freelisted by particular groups: maxima, minima, median (thick line inside a box), 25 and 75 percentile (borders of the box).** Outliers are marked with a small circle.

**Figure 4:**
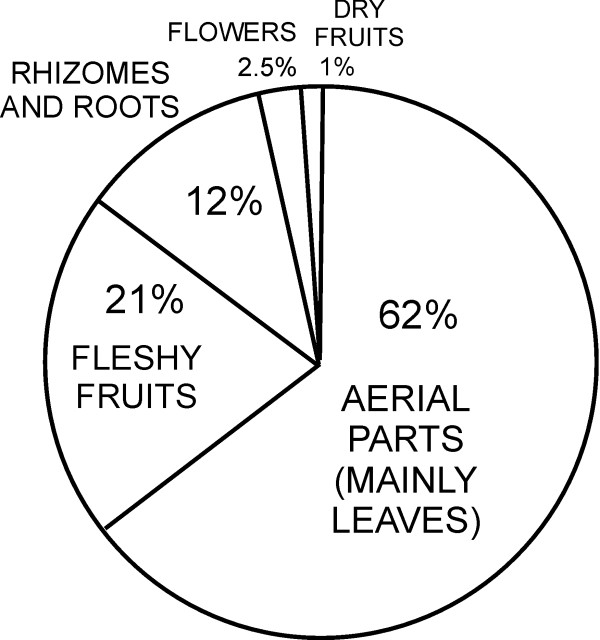
Use categories in the listed species.

The mean number of freelisted wild foods (Figure 3) was higher in the Houzhenzi valley (24.8 and 17.6 respectively; Mann-Whitney U test, p?<?0.05). A similar trend was observed in all the three categories: wild vegetables (mean 17.5 and 11.5 respectively), fruits (5.9 and 5.1) and fungi (1.9 and 1.0), though the difference was significant only for the vegetables. In both valleys people listed many species of wild vegetables, and few species of fruits, while they struggled to list edible fungi (Figures 3, 4).

**Figure 5:**
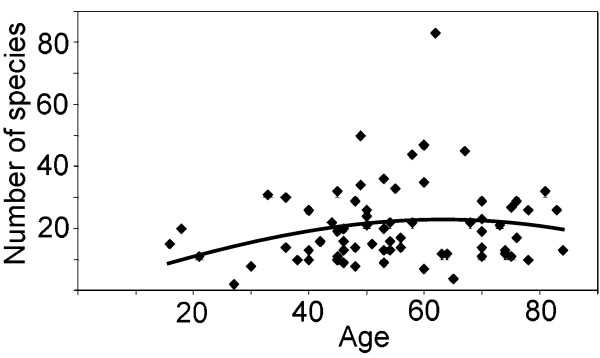
**The relationship between the listed number of edible species and age.** A polynominal curve explains the relationship better than a linear model (-0.00665?×^2^?+?0.8327?×-2.807, R^2^?=?0.048, p?=?0.18).

The overall number of species was significantly positively correlated with the male gender (Spearman rho?=?0.29, p?<?0.05) and Houzhenzi valley (rho?=?0.28, p?<?0.05). There was a small correlation with age, but it was not significant. Species number versus age relationship was better explained by a polynominal curve (-0.00665?×^2^?+?0.8327?×-2.807, R^2^?=?0.048, p?=?0.18; Figure 5) with maximum values for people in their early sixties, though the fit was still not significant (p?=?0.18).

As many as 42% of the folk taxa are typical woodland species and only 21% are ruderal species from fields and field edges. The remaining taxa come from forest edges, forest clearings, thickets, grasslands and water margins, thus in practice over half of the species come from woodland ecosystems. This high proportion of forest species and low proportion of ruderals is even more pronounced in Houzhenzi (44% and 14% respectively, compared to 41% and 25% in Dali, a not significant difference: Chi Squared test, p?=?0.07). Although a large proportion of plants are woodland taxa, it is the herbs that dominate in the species list (69%), with shrubs, trees and vines playing a minor role (15%, 13% and 3% respectively).

### Wild vegetables

**Figure 6:**
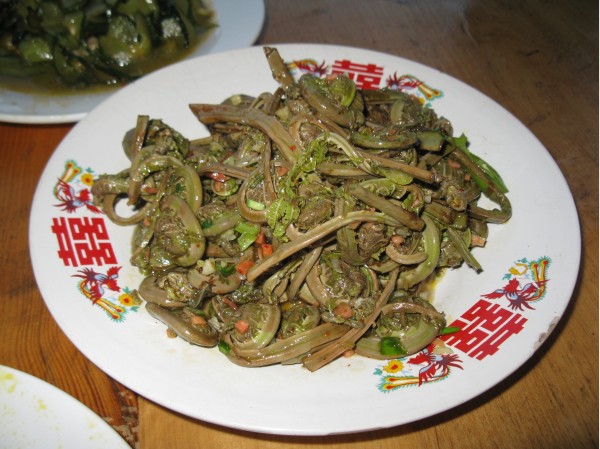
***Matteucia struthiopteris *shoots, boiled, strained and sprinkled with oil.**

**Figure 7:**
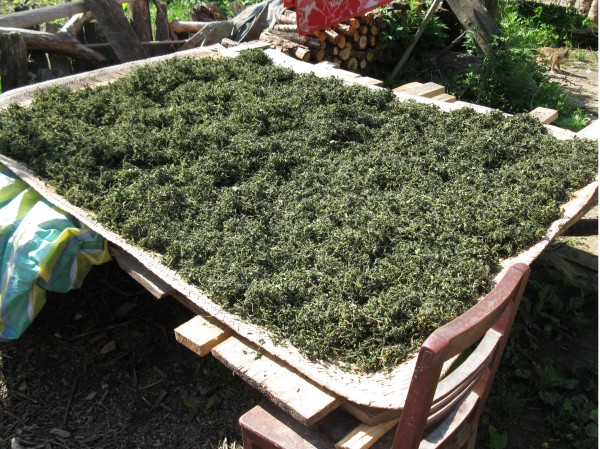
**Drying wild vegetables *(Staphylea bumalda) *in Houzhenzi in early June 2011.**

**Figure 8:**
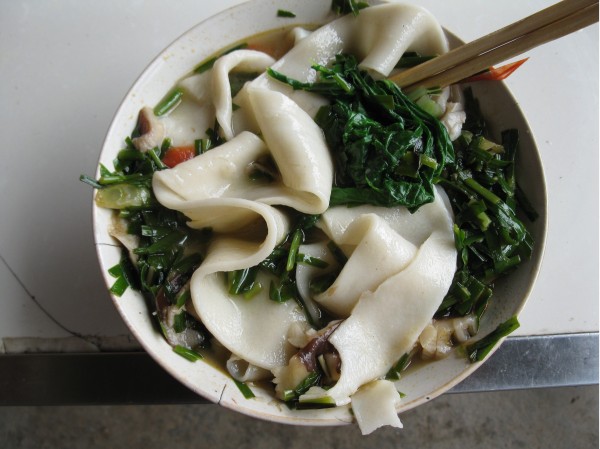
Adding wild vegetables to noodle soup is another common form of utilizing the plants.

Wild vegetables are the most important wild food category collected (Figures 3, 4 and 6, 7, 8, Table 3). This was expressed both by the fact that they constituted around two thirds of the species lists and that people most eagerly talked about them. They are also the only category of wild food stored for winter. Drying wild vegetables is a very common preserving technique (Figure 7). Households dry between 1-5 species each year, usually a few kg of dry shoots and leaves, but some households who host tourists can dry even a few times more. Formerly, wild vegetables were lacto-fermented, but now this is done very rarely.

The number of wild vegetables listed was positively and significantly correlated with place (Houzhenzi versus Dali), male gender and age (Spearman rho equals 0.35, 0.27 and 0.26 respectively), however these are relatively low correlation values.

The ruderal species are collected near homesteads. Their growth is often promoted by sparing them from being sprayed with glyphosate (e.g. *Chenopodium album, Hemerocallis* spp.). Some forest species are harvested up to 5 km from the villages, up to the altitude of 1800 m a.s.l. At even higher altitudes, wild plants are only harvested while collecting medicinal herbs, which grow even higher.

Wild vegetables are frequently eaten in all meals, mainly as a side dish (*cai*). The commonest preparation technique is boiling, then straining and sprinkling them with some oil in which Sechuan pepper, garlic, and sometimes ginger, have been fried. This is a side dish, called *liang ban,* accompanied by home-made wheat bread (*bing*), rice or other stir-fried foods. Frequently wild vegetables are also put into broad, home-made noodles served in a spicy and sour soup. They are also, rarely, lacto-fermented. Wild vegetables are also sold in all the local restaurants and every agritourist farm has them on their menu.

### Fruits

Wild fruits are little appreciated. They are occasionally gathered for fun by children or grown-ups going to the forest. They have never been stored or dried, and are not incorporated in any dishes by anyone, apart from dried *Schisandra* and *Akebia* fruits (the latter more rarely), used medicinally. There was no significant correlation between the number of fruits listed and age, location or gender.

### Fungi

Few fungi species are used in both valleys. Most people never go to the forest with the purpose of collecting mushrooms, apart from going to collect cultivated *Auricularia* sp. and *Lentinula edodes* grown on piles of logs located in the woods. There was no significant correlation between the number of fungi listed and age, location or gender, though the difference between the male and female groups was bordering on significance level (Mann-Whitney U test, p?=?0.08, Spearman rho?=?0.20).

The most frequently mentioned mushrooms in both valleys are *Cantherellus cibarius,* an unidentified *Agaricales* (called *banlijun*, i.e. “chestnut mushroom”), *Ramaria* spp. treated by locals as one folk taxon and *Grifola umbellata,* (its sclerotia are additionally collected for medicinal purposes). More than half of the respondents had never collected wild fungi in the forest.

### Famine plants

All the older informants were asked about plants eaten during the severe food shortages that plagued China until the last case of famine in 1958-60. However this revealed only a few “famine” plants, as the respondents stated that they rather ate the same wild plants but in larger quantities. Underground organs of plants were particularly eagerly sought after: the rhizomes of *Pueraria lobata, Pteridium aquilinum, Polygonatum* spp., S*inacalia tangutica,* the bulbs of *Lilium giganteum* and other *Lilium* species. Nowadays the consumption of underground organs of wild plants has practically ceased. Many respondents also mentioned using the leaves of *Abelia engleriana* to make a special dish called *shenxiandoufu* (i.e. *fairy tofu*). According to legend, during times of famine a fairy/wizard/holy man passed through the area and taught people how to make a special famine tofu with this plant, which saved people from starvation. The bark of *Ulmus* spp. was used to make famine bread, however not in the 1958-60 famine, but in the previous 1940s famine (before the Liberation), which in this area is remembered as being more severe.

## Discussion

It was already pointed out by Kang et al. [13] that the large number of wild greens used in this valley is one of the highest recorded on such a small scale in ethnobotanical studies. Only Zou et al. [9] recorded more, noting the use of 335 taxa of wild vegetables in 10 villages of Hunan, however the latter study was carried out in a larger and more heterogeneous area. Ghorbani et al. [11] recorded the use of 173 wild food plants from 485 informants of four ethnic groups of Naban valley of Xishuangbanna (tropical area of south China), out of which only around a third were wild greens, in contrast to our study where they dominate. However, Ghorbani’s study concerned an area, which was very heterogeneous in terms of elevation, inhabitants and vegetation. The average number of wild food plants listed by one informant was only around 10 species, whereas in this study we documented twice as many taxa per person.

The presented list is also much longer than the lists of wild food plants reported in previous studies from the Qinling Mountains, east from our study area, in the south of the Huxian county [38,39]. Although there was a partial overlap in the species lists (Table 3), the differences show a high geographical diversity of wild vegetable use. Some of these differences may come from differences in habitats, some from cultural choices, and some from the fact that probably only the commonest wild vegetables were reported by Ma et al. [38,39].

It was already pointed out by us in the previous paper [13] that knowledge of wild vegetables in China is additionally encoded in the language (many wild vegetables have *cai,* i.e. vegetable), in their name. In our study it was 39 taxa (a third of the wild vegetables recorded, mainly the most commonly used ones). Another factor may nowadays help to preserve the knowledge of wild vegetables in China. It is the commodification of wild vegetables by involving them in tourism. Nowadays, nearly every national park in China has local restaurants of wild vegetables (often called “mountain wild vegetables” to emphasize their “naturalness”) (e.g. [9,11,13]). This is part of a broader process of trying to promote or create local attractions in rural China with the hope of drawing the attention of tourists [40]. A similar process occurs in Europe, where wild foods are incorporated in haute cuisine and in revitalized regional dishes [41].

It must be pointed out that the number of wild foods used in many parts of China, in its species-rich parts outside the areas of intense agriculture, is probably an example of an area utilizing the largest number of plant species available to human populations. This huge list of plants is mainly made up by wild greens. This attitude was named by Łuczaj [42] as *herbophilia*, and here in China it takes its most extreme form. The number of wild food plants used in the studied valley comparable to the lists of edible plants recorded on a country scale in Europe (e.g. [43-49]). The utilization of such a large number of greens may also be found in some communities in other countries of Eastern Asia, e.g. Japan, Korea, Vietnam and Thailand [50-57], as well as in some parts of India and Africa [54,57]. The number of wild food plants used by the studied communities in the Qinling Mountains is similar to that used by the few communities in the world previously reported as using the highest numbers of wild food plants species (between 171 and 252), i.e. Igbo ib southern Nigeria, Dalit in Andhra Pradesh or Karen in NW Thailand ([57] after [54]).

In many tropical and subtropical areas the choice of species is limited by the fact that primary tropical vegetation has thick leathery leaves and mainly ruderal plants are eaten. In the mountains of China the choice of edible species is increased by two factors:

1. large elevation differences enabling easy access to different vegetation zones;

2. deciduous vegetation with many forest understory perennials with delicate leaves and buds.

In our study around half of the wild vegetables come from the forest. This is in contrast with other ethnobotanical studies showing that human populations, even in wooded areas, tend to over-utilize the ruderal flora [14,58-60]. On the other hand the results of this study are very similar to the data on the use of wild food plants by the Karen ethnic group in the forests of NW Thailand, for whom wild forest vegetables also constitute a major part of the wild plants consumed [55]. Interestingly, the large diversity of wild vegetables (also those which are typical woodland species) used in Eastern Asia remains in stark contrast with the extremely low number of wild vegetables used in the Amazon in similar environments [61].

What is interesting is the large domination of wild greens over fruits and fungi. The list of mushrooms is for example shorter than the number of mushroom species used locally in Poland [62] or Mexico [63]. The lack of interest in wild edible mushrooms in the studied area is puzzling, as China is sometimes regarded as a mycophilous part of the world [64,65]. It may arise from the easy access to the cultivated *Auricularia* and *Lentinula* mushrooms and extremely steep terrain, making foraging for fungi difficult. In Yunnan, famous for edible fungi, the hills and mountains are often less steep (ŁŁ, personal observations). Collecting mushrooms requires more walking to find them than in the case of wild vegetables whose location is more permanent. Our results encourage further research into the knowledge and use of edible fungi in rural areas in China.

### Do men know more?

In the Qinling mountains the collection of wild food plants is the domain of both sexes. On the other hand it must be noted that men slightly outperform women in listing slightly more species of wild vegetables, and many more species of fungi (Table 3). These results may be caused by the fact that a larger proportion of men get involved, regularly or occasionally, in collecting medicinal plants for sale. As they make long trips into higher elevations they acquire a vast knowledge of local woodland flora. This may explain the larger number of species listed, even though it may be the women who spend more hours collecting wild plants. This slight domination of men in this domain is quite unusual, as in many countries it is the women who are the main holders of knowledge on wild vegetables (e.g. [42,66,67]), and even fungi [68]. This unusual tendency was also noted in Poland, where it is the men who are more involved in collecting wild fungi [62].

## Conclusions

The study yielded one of the longest lists of wild food plants used locally ever recorded in ethnobotanical studies. In both the studied valleys, wild vegetables are still widely used throughout the year and preserved for winter. In the more developed Dali valley people use slightly fewer wild vegetables than in the Houzhenzi area. Although usually only a few species are collected in larger quantities, knowledge about these plants is still very alive. On the other hand the community shows relative indifference to wild fruits and fungi, which are rarely collected, and only as an additional activity. There were small differences in the knowledge of wild foods among the members of different age groups and between men and women.

The results of this study show that further in-depth ethnobotanical research is needed to determine patterns in wild food plant and fungi use in different parts of China, as locally these patterns may be extremely variable. Also more research recording age and gender differences is needed.

## Competing interests

The authors state that they have no competing interests.

## Authors’ contributions

All the authors took part in the interviewing process in the field, voucher specimen preparation and data processing, and read the final version of the manuscript. ŁŁ designed the study, YK organized the expedition, led the interviews and identified most taxa. ŁŁ and YK wrote the article together. All authors read and approved the final manuscript.
